# Sexual Health and Well-Being in Adults With Congenital Heart Disease: A International Society of Adult Congenital Heart Disease Statement

**DOI:** 10.1016/j.jacadv.2023.100716

**Published:** 2023-11-18

**Authors:** Lorna Swan, Jonathan Windram, Luke Burchill, Laila Akbar Ladak, Leigh C. Reardon, Beatriz Fernandez, Roni M. Jacobsen, Maggie Simpson, David Harrison, Liza Morton

**Affiliations:** a Scottish Adult Congenital Cardiac Service, Golden Jubilee NHS Hospital and University of Glasgow, Glasgow, United Kingdom; b Mazankowski Heart Institute, University of Alberta, Edmonton, Canada; c Mayo Clinic Adult Congenital Heart Disease Program, Rochester, Minnesota, USA; d The Aga Khan University School of Nursing and Midwifery, Karachi, Pakistan; e Ahmanson/UCLA Adult Congenital Heart Disease Center, UCLA Children’s Heart Center, Los Angeles, California, USA; f Division of Cardiology, Instituto Mexicano del Seguro Social, Mexico City, Mexico; g Division of Cardiology, Departments of Internal Medicine and Pediatrics, University of Colorado School of Medicine, Denver, Colorado, USA; h Cardiology, NHS Greater Glasgow & Clyde, Glasgow, United Kingdom; i Department of Medicine, University of Colorado, Denver, Colorado, USA; j Glasgow Caledonian University, Glasgow, United Kingdom

**Keywords:** adult congenital heart disease, advocacy, contraception, gender, sexual health

## Abstract

As health care outcomes improve the priority for those living with adult congenital heart disease have changed to a more holistic focus on quality of life and well-being. Although health care has embraced this, there are still areas where there is a deficit in advice, allyship, and advocacy. One of these deficits is in the area of sexual health and well-being. A healthy sexual life has a myriad of physical and psychosocial benefits. However, individuals with adult congenital heart disease may have significant barriers to achieving well-being in this aspect of their lives. These barriers and their potential solutions are outlined in this paper.

## Highlights

• Sexual dysfunction is common in individuals living with congenital heart disease.
• The adult congenital heart disease team should provide an open inclusive environment to discuss sexual health.
• Psychological and physical barriers to having a fulfilling sexual life are often treatable.
• Further education and research are needed to improve this aspect of holistic adult congenital heart disease care.

**Figure 1:**
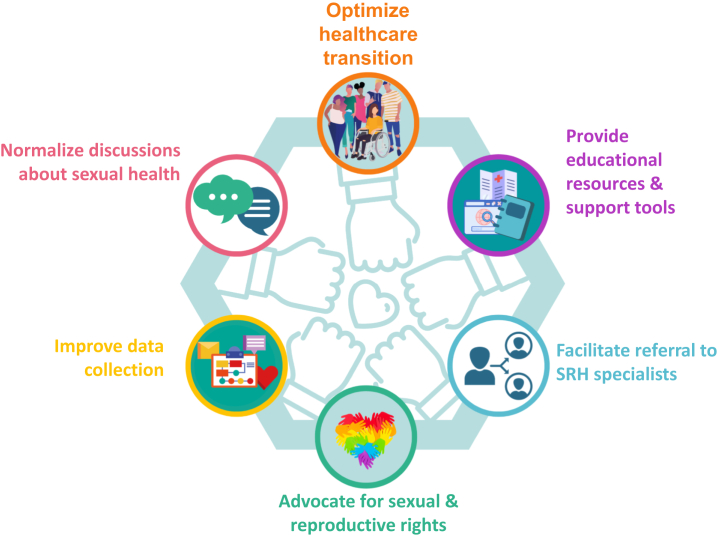
Lived Experience Quotes

The lifestyle implications of living with a congenital heart lesion are routinely discussed in specialist adult congenital heart disease (ACHD) clinics.^1^ There is, however, one lifestyle issue that is often overlooked: advice and support for a fulfilling sexual life. This is not just a deficit in ACHD care but is particularly important for young individuals with a complex, lifelong cardiac condition. A healthy sexual life has a myriad of health benefits including improved immune functioning, pain control (Figure 1), reduced risk of depression/anxiety, and enriched social integration.^2^, ^3^, ^4^ A reduction in mortality has also been reported.^5^ The clinical team, therefore, has an important role in minimizing the barriers patients experience in achieving sexual well-being.

All health care providers (HCPs) should have knowledge in sexual health, sexual orientation, and gender as it relates to health. There should be a high level of sophistication in the use of language and inclusivity. Sexual health is a complex interaction of health, beliefs, culture, values, and emotions. For some individuals with ACHD and HCPs, previous sexual trauma may also cloud this issue.^6^ The HCP should be mindful of this and recognize when patients need additional support beyond their own expertise.

The term sexual dysfunction is defined in this paper as the inability to experience sexual arousal or to achieve sexual satisfaction under appropriate circumstances. This dysfunction includes difficulties related to sexual desire, arousal, orgasm, and sexual pain.

The aim of this paper is to outline the psychological and physical barriers to sexual well-being for those living with ACHD. The paper will also signpost readers to resources that may help in the discussion and treatment of these challenges. (Table 1). The health care environment has usually framed health through a heteronormative lens, and LGBTQIA+ issues have been largely overlooked.^7^ The health care team needs to be mindful of this bias.

**Table 1:** Resources for Individuals With ACHD and Health Care Providers

Name	Summary	Link
Heart disease (general)		
Sexual activity and heart patients: a contemporary perspective	Risk stratification and recommendations for sexual activity in those with heart conditions	https://pubmed.ncbi.nlm.nih.gov/26690295/
CDC: taking a sexual history	A guide for health professionals for taking sexual history, factsheet	https://www.cdc.gov/std/treatment/sexualhistory.pdf
AHA scientific statement	Evidence based recommendations regarding sexual activity in patients with cardiovascular conditions	https://www.ahajournals.org/doi/10.1161/cir.0b013e3182447787
Sexual activity and cardiovascular disease		
Sexual counseling for individuals with cardiovascular disease and their partners	Evidence based recommendation for sexual counseling in patients with cardiovascular disease and their partners	https://www.ahajournals.org/doi/full/10.1161/cir.0b013e3182447787#:~:text=In%20patients%20with%20unstable%20or,is%20stabilized%20and%20optimally%20managed
Domestic abuse	Responding to domestic abuse; a resource for health professionals	https://www.ahajournals.org/doi/full/10.1161/CIR.0b013e31829c2e53
Sexual advice association	Factsheet and self-assessment for those with heart conditions and erectile dysfunction	https://sexualadviceassociation.co.uk/wp-content/uploads/2016/02/Sex-and-the-Heart.pdf
BHF resource on erectile dysfunction and heart disease	Magazine from the British Heart Foundation. Advice and stories from individuals with heart conditions	https://www.bhf.org.uk/informationsupport/heart-matters-magazine/wellbeing/erectile-dysfunction
Up-to-date: Sexual activity in patients with cardiovascular disease	Evidenced based recommendations regarding sexual health and on sexual activity in patients with cardiovascular conditions Limited access required institutional login or paid access	https://www.uptodate.com/contents/sexual-activity-in-patients-with-cardiovascular-disease https://www.uptodate.com/contents/sexual-problems-in-females-beyond-the-basics https://www.uptodate.com/contents/sexual-problems-in-men-beyond-the-basics
United Nations Population Fund sexual and reproductive health	Overview and resources around advocacy for sexual and reproductive health from a global perspective	https://www.unfpa.org/sexual-reproductive-health
World Health Organization Sexual and Reproductive Health Research Team	Overview and resources around advocacy for sexual and reproductive health from a global perspective	https://www.who.int/teams/sexual-and-reproductive-health-and-research-(srh)/overview
Sexual health concerns in patients with cardiovascular disease	AHA paper focusing on common sexual activity concerns in patients with heart disease	https://www.ahajournals.org/doi/10.1161/CIRCULATIONAHA.113.004846
Heart-failure.net	Online portal for those with heart failure. Education resources and patient stories	https://heart-failure.net/
The sensuous heart S Cambre	Free online book focusing on the most common questions asked regarding sexual life after having a heart surgery or a MI	https://issuu.com/pandh/docs/thesensuousheartbook?e=1149653/9678051
American Health Association Statement of cardiovascular health for LGBTQ individuals	Overview of cardiovascular health issues for LGBTQ individuals	https://professional.heart.org/en/guidelines-and-statements/guidelines-and-statements-search
Identifying nonrecent childhood abuse	Patient and carer information on the signs of nonrecent abuse	https://www.ahajournals.org/doi/10.1161/CIR.0000000000000914
Resources for those with learning difficulties	Series of easy read downloadable leaflets on relationships and sex	https://www.mencap.org.uk/advice-and-support/relationships-and-sex
Adult congenital heart disease specific		
Relationships & sexuality for people with heart disease	Information for parents of children with congenital heart disease	https://www.aboutkidshealth.ca/article?contentid=1710&language=english
Healing hearts and minds, T Livecchi. L Morton, Oxford Press	Recently published book focusing on a psychological toolkit for those with CHD and their families	https://www.aboutkidshealth.ca/Article?contentid=1710&language=English
Intimacy & pulmonary hypertension	Booklet from the Pulmonary Hypertension Association UK focusing on how PH might affect sexual well-being and relationships	https://www.phauk.org/product/intimacy-and-ph/
AHA scientific statement. Psychological outcomes and interventions for individuals with congenital heart disease	Summary of the psychological outcomes and interventions in adults with CHD	https://www.ahajournals.org/doi/10.1161/HCQ.0000000000000110

ACHD = adult with congenital heart disease; AHA = American Heart Association; CHD = congenital heart disease; MI = myocardial infarction.

## Sexual behavior in people living with ACHD

### Sexual health literacy

Previous studies addressing sexual health in ACHD have focused on knowledge deficits.^8^ Despite different cultural beliefs and counseling practices around the world, these knowledge gaps are encountered internationally. Most studies describe health literacy around pregnancy and contraception.^9^ Women with congenital heart disease (CHD) described their knowledge in these areas as 5 out of 10; they describe their knowledge around sexuality as a median of 3.5 out of 10.^10^ Across multiple studies, 35 to 59% of women with CHD could not recall being counseled about these subjects.^11^^,^^12^

Beyond pregnancy and contraception even fewer studies exist. In Korea, ACHD patients scored lower on the Knowledge in Safe Sex Practice scale, especially those with complex cyanotic conditions.^13^ Burstrom et al. identified that only 70% of adolescents with CHD could correctly identify whether sexual activity (SA) could worsen their heart condition.^14^ Only 22 to 50% knew that SA was not a risk factor for endocarditis.^15^^,^^16^

### Sexual beliefs and practices

Data on SA, relationships, and safe sex practices is scarce and not uniform in its findings. Some studies suggest that people with ACHD exhibit similar sexual practices to their healthy peers. In an interview series, people living with CHD showed no evidence of avoiding SA and overall engaged in “normal” dating with the exception of those with physical limitations.^17^ Other studies endorsed these findings showing no difference in the likelihood of being sexually active, age of first intercourse or rates of sexually transmitted infections.^18^ This is consistent with data on adolescents with other chronic conditions. Moons et al. found that individuals with ACHD spent less time “worrying about [their] sex life” and “not enjoying having sex”. A subset did, however, report anxiety and distress.^19^

Contrasting studies suggest avoidance and/or distress related to dating and sexual experiences. However, once a relationship is established, it is often fulfilling. Other studies describe people with CHD having fewer romantic partnerships.^20^^,^^21^ But again, people with ACHD, once in a relationship, perceived this as more satisfying. Insecurities regarding body image and distress during sex are also reported.^22^ Fry et al. encountered lower rates of ever having had sexual intercourse among adolescents with CHD.^23^ Reid et al. also described this, but also reported that a high proportion were engaged in potentially risky sexual behaviors, including having multiple partners, inconsistent condom/contraceptive use, and/or concomitant use of alcohol and drugs.^24^

The mixed data surrounding sexual behaviors in the ACHD population may reflect varied cultural or personal beliefs regarding sexuality that are difficult to generalize. It may also reflect the heterogeneous nature of ACHD lesions.

Many individuals with ACHD are happy and fulfilled without having an active sex life. They would not consider themselves asexual (although some are) but do not have any desire to increase this part of their lives. As in all areas of health care, assumptions should not be made about individuals’ preferences or wishes.

## Psychological factors affecting sexual function

For some, living with congenital heart disease can adversely affect intimacy. While everyone’s experience is unique and many people with CHD enjoy a healthy sex life, many encounter difficulties including orgasmic dysfunction, erectile dysfunction, intercourse dissatisfaction, lower sexual desire, and an increased incidence of dyspareunia. Psychosocial barriers that may contribute to these findings include impaired body image and lower self- and sexual esteem.^25^ Fear of death, illness, or experiencing cardiac symptoms during sexual intercourse is also limiting for others, as are concerns about pregnancy, fear of rejection and anxiety around starting or maintaining an intimate relationship.^26^ Chronic illness can also alter the dynamics in a relationship when the partner takes on the role of a caregiver.

### Medical trauma

Adverse childhood experiences, including serious childhood illness, have been associated with a negative impact on sexual health.^27^ Individuals need to feel psychologically safe for intimacy and healthy relationships. Living with a serious, lifelong medical condition and facing repeated exposure to medical and body trauma can affect this. Individuals with CHD may have to cope with uncomfortable physical symptoms, an uncertain prognosis, and exposure to medical procedures. This often includes childhood experiences of cardiac catheterization, devices such as pacemaker or implantable cardioverter-defibrillator implants and cardiac surgery. Cardiac events can also be abrupt and unexpected. Difficulty accessing specialist care can further impact feelings of psychological safety.^28^ Other specific aspects of care lead to feelings of disempowerment, for example, backless hospital gowns^29^ and clinical holding of children for medical procedures (iSUPPORT 2022). The resulting sense of threat and post-traumatic stress can adversely affect emotional regulation and healthy attachment. Conflicting medical advice can exacerbate sexual health concerns, particularly regarding recommendations around restricting physical activity or pregnancy risk. Unhelpful health beliefs can be alleviated by HCPs validating and exploring concerns, acknowledging and updating any mixed messaging, educating individuals about red flag symptoms, and linking with sexual health teams.

### Body image and self-esteem

Low self-esteem is a negative image of oneself that is global, persistent, and enduring often rooted in negative early life experiences. Low self-esteem is correlated with vulnerability to low mood, anxiety, maladaptive coping strategies, and poorer sexual health.^30^ In turn, sexual health impacts self-esteem and identity formation, especially during adolescence.

While it should not be assumed that low self-esteem is an inevitable consequence of living with CHD people living with chronic illnesses are often marginalized, pitied, and even blamed for their condition. This includes being treated differently, facing hidden barriers and stigma that can result in feelings of shame and discrimination.^31^

Body image concerns can present a barrier to sexual health. While CHD is generally considered an ‘invisible’ disability, some people with CHD have scarring, a visible cardiac device (eg, pacemaker), short stature, cyanosis, pectus, scoliosis, or lack of ability to increase muscle mass. Men, in particular, face cultural expectations to be strong, fit, and muscular. Feeling different can negatively impact the development of sexual confidence. Parental overprotection and messaging that you are a ‘miracle baby’ can further contribute.^32^

When CHD is not readily visible, the individual with CHD can have concerns about when and how to reveal it in a new relationship. More so if this has led to rejection in the past. This can result in people with CHD masking symptoms, finding it difficult to trust others, concealing concerns, and overcompensating an attempt to ‘fit in’. This can lead to an ongoing negative cycle related to body image.

Physical activity contributes to the development of positive body image and reduces social physique anxiety. Historically, many people living with CHD were advised to restrict physical activity. Since sports are often a rite of passage, especially for adolescents, any loss of such opportunities can lead to missed opportunities to develop lasting friendships while impacting self-esteem.

Serious illness is known to negatively affect body confidence. Living with a chronic health condition and facing periodic health crisis can impact a sense of trust in one’s body.^33^ Cardiac rehabilitation programs for people with CHD may go some way to rebuild body confidence and fitness.

### Mental health difficulties

Psychosocial barriers including feeling of loss (eg, of a ‘normal’ childhood), parental overprotection, impact on relationships, scarring and body image, issues having and raising a family, and an increased prevalence of neurological sequelae.^34^, ^35^, ^36^ Shared disadvantages with other minority groups including discrimination and ableism, disparities in income, education, employment, and underrepresentation in the media and politics.^37^^,^^38^

All of this considered, perhaps it is not surprising that the lifetime prevalence of depression, anxiety, and post-traumatic stress disorder for adults with CHD is 50%.^39^ These, in turn, can cause sexual dysfunction that can also be aggravated by psychotropic medications. When this is recognized, referral to psychological and psychosexual therapy can be very beneficial. Improved screening, recognition, and support for underlying mental health difficulties are vital to sexual health.

### Sexual abuse risk assessment

It is important for HCPs to be aware that people with disabilities are at significantly increased risk of sexual violence and intimate partner violence. This increased risk is also associated with poorer health status and limited access to health care.^40^

### Positive psychological health

Protective factors for psychological health include less parental overprotection, more affection during childhood, social acceptance ^41^ and disability pride.^37^ Many people who live with a disability report a high quality of life often in contrast to how they are perceived by others, known as the “disability paradox”.^42^ Well-being is complex, and every person with CHD is unique, but promoting protective factors and mitigating risk factors is likely to optimize health.

Many people with CHD have positive adaptation such as having more meaningful relationships and increased resilience. This post-traumatic growth can be built by promoting empowerment and health literacy. It is essential that HCPs are skilled in confidently discussing sexual health with patients and create an environment conducive to them sharing their concerns.

## Physical factors affecting sexual function

Sexual dysfunction affects 28% of people living with CHD.^43^ Risk factors include those with exercise intolerance (Central Illustration), arrhythmias, impaired functional status, and the use of certain cardiac medication (eg, digoxin and spironolactone).^44^ Table 2 lists a more complete list of medications that may affect sexual function. On the other hand, optimal medical treatment may improve sexual function such as the use of sacubitril/valsartan in heart failure.^45^

**Central Illustration:**
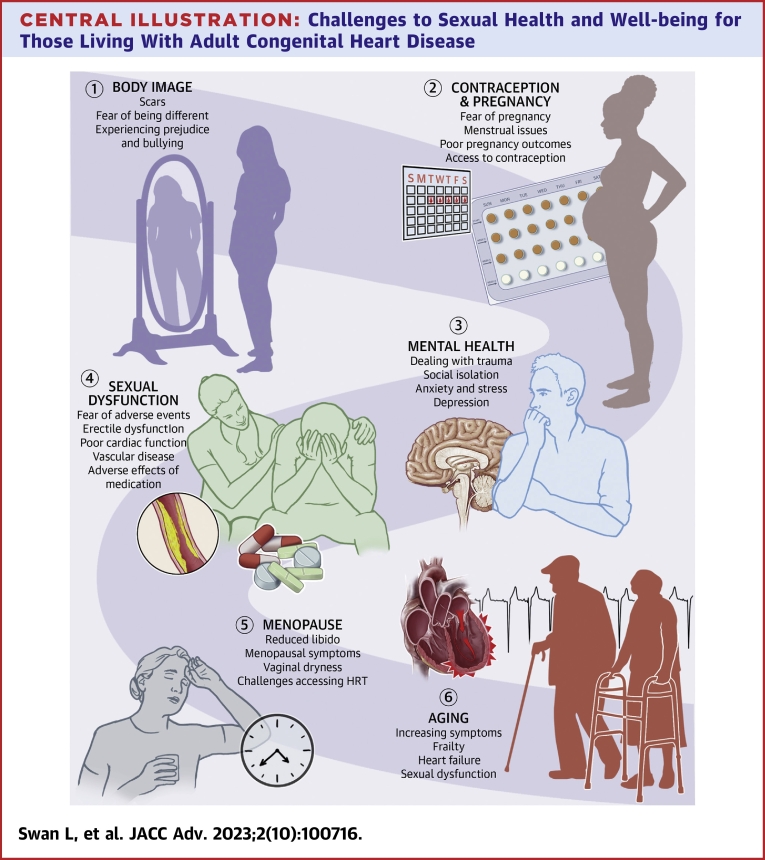
Challenges to Sexual Health and Well-being for Those Living With Adult Congenital Heart Disease

**Table 2:** Cardiac Drugs That May Be Associated With Sexual Dysfunction

Type of Sexual Dysfunction	Drug
Erectile dysfunction	Angiotensin receptor blockers, beta-blockers, clonidine, calcium-channel blockers, diuretics, digoxin, methyldopa, spironolactone, statins, thiazides
Vaginal dryness	Anticholinergics, antihistamines, beta-blockers, calcium-channel blockers, diuretics, spironolactone
Reduced libido	Diuretics, metoprolol, spironolactone
Priapism	Doxazosin, warfarin
Impaired ejaculation	Doxazosin
Testicular dysfunction (elevated serum gonadotrophins) (chronic epididymitis)	Amiodarone

Cardiac drugs that improve ventricular function/symptoms may improve sexual function.

Erectile dysfunction is common, with an incidence between 10% and 43% in those with ACHD. ^26^^,^^44^ Age >40 years, moderate to high CHD complexity, diabetes, heart failure, diuretic use, anti-arrhythmic treatment, antidepressants, and being single were associated with risks by univariate analysis. The independent predictors were anxiety to participate in SA, age and diagnosis of a psychiatric disorder.^46^ Another single study demonstrated a 3-fold risk for erectile dysfunction in young adult CHD males on beta blocker and/or angiotensin receptor inhibitor therapy ^47^ but this finding has not been consistent in other studies. Endothelial dysfunction has also been implicated in sexual dysfunction.

Menstrual dysfunction affects 83% of young adult women with CHD although only 20% seek medical support.^48^ This includes menstrual cramping, irregular menses, and heavy menses. Delayed menarche is also common especially in those with complex CHD such as those with a Fontan circulation. There is a 3-fold risk of menorrhagia in women on anticoagulants, and this should be pro-actively discussed when prescribing these agents.

Given the high prevalence of sexual dysfunction in individuals with CHD clinicians should proactively inquire about symptoms. The cardiac specialist’s role will include a full cardiovascular assessment focusing on possible cardiac cause. Specific interventions include reviewing medication—for example, optimizing heart failure medication—and, if possible, changing medications known to be associated with sexual dysfunction. In women presenting with menorrhagia, the requirement and control of anticoagulation should be reviewed. In older patients with new erectile dysfunction, the possibility of superimposed atherosclerotic disease should also not be forgotten. In the younger patient with a Fontan circulation, scrotal edema and hydroceles may be signs of Fontan failure. Sexual dysfunction may also be a sign of health deterioration and overall frailty, and in these circumstances, it may be warning of future adverse events.

The importance of lifestyle modification is also key in optimizing sexual health. Normalizing body mass index, exercising, and reviewing blood pressure, blood glucose, and lipid profile should be part of this assessment. However, there should not be a barrier to patients being referred to urology, gynecology, and endocrine specialists. Comprehensive expert assessment is required before sexual dysfunction is attributed to the cardiac condition per se, and even then, patients should have access to the full range of treatment options.

## Cardiovascular risks of sexual activity

SA can be a cause of worry for individuals with heart disease. Several hemodynamic changes occur during SA, reaching a maximal effect upon orgasm. It has been determined that SA during the preorgasmic phase is equivalent to 2 to 3 metabolic equivalent of tasks (METs) of exertion, increasing to 3 to 4 METs during orgasm. This would be the equivalent of walking briskly on the flat or climbing stairs. With the data available, it can be concluded that sexual intercourse results in a modest but brief increase in myocardial oxygen demand.^49^

Though several studies look at the cardiovascular response of SA, these are limited by methodology. Initial studies were performed in the laboratory setting.^50^^,^^51^ These studies suggested that SA had a high workload with peak heart rates of 140 to 180 beats/min and a mean increase in blood pressure of 80/50 mm Hg during orgasm. Subsequent studies of married heterosexual couples who were monitored in their own homes revealed less dramatic changes.^52^^,^^53^ In 1 study, mean heart rate at the time of orgasm was 117 beats/min. A mean blood pressure of 162/89 mm Hg was estimated from equivalent levels of exercise, similar to values observed in other studies.

The risk of sudden cardiac death during SA is small with SA implicated in only 1.5% of cases. In an autopsy study, the annual incidence of sudden cardiac death during SA was 0.16 per 1,000 autopsies for women and 1.9 per 1,000 autopsies for men.^54^ Lee et al. found that only 1% of deaths occurred during SA.^55^ Most cases occurred in extramarital relationships, but the authors postulated that this might be a reporting bias.

The relative risk of having a myocardial infarction during SA is low at 2.7, which is reduced further in those who are physically active.^56^ In 536 individuals who suffered from a myocardial infarction, only 0.7% reported having SA within an hour of the development of their symptoms.^57^ Those who suffered angina during SA all reported angina during other forms of exercise. Therefore, exercise testing can be helpful, and those who achieve MET level of 3 or greater without angina have a low likelihood of suffering from angina during SA.^58^

There is little data regarding the risks of SA specific to the ACHD population. Vigl et al^59^ surveyed women with CHD and revealed that 9% reported symptoms during SA ranging from dyspnea, palpitations, fatigue, and syncope. Those with severe lesions, cyanosis, or worse functional status were more likely to report symptoms. Men reported similar rates of dyspnea (9%), palpitations (9%), and chest pain (5%), which were again most common in those with a lower functional class.^26^

SA is safe for most individuals with CHD. This is less clear for individuals with cyanotic heart disease, pulmonary hypertension, severe left ventricular outflow obstruction, heart failure, or those with ischemic chest pain. Those with a significant hemodynamic lesion who experience symptoms during SA should alert their HCP to the fact that they are symptomatic at low levels of exertion, which likely merits further investigation. Extrapolating from the data of individuals with coronary artery disease, the use of exercise testing may be informative. In addition, exercise and cardiac rehabilitation may reduce the risk of sex-related cardiac events.

### LBGTQIA+ individuals

Lesbian, gay, bisexual, transgender, queer, intersex, asexual, and + (LGBTQIA+) individuals with CHD represent a growing demographic of patients who need thoughtful alliances with ACHD providers to avoid medical misadventures and to enable them to live healthy and fulfilling lives. Though LGBTQIA+ people are often grouped together, subgroups have distinct considerations when interfacing with congenital heart specialists. Surveys and studies demonstrate an increasing number of individuals who identify as LGBTQIA+ (Gallup, CDC Vital Statistics, Williams Institute) over the past decade. As of 2020, 7% of the population and as high as 20% of Gen Z individuals (born between 1997 and 2002) identify as LGBTQIA+ with approximately 0.6% of the population identifying as transgender.^60^ These studies have a significant bias because they were conducted in western industrialized nations where there is growing social, cultural, and legal acceptance of LGBTQIA+ people. In many societies, LGBTQIA+ individuals still experience marginalization, discrimination, persecution, and violence.

Despite recent advances, such as the 2020 AHA statement, there is still limited research into the health determinants of this population.^61^ This is despite the knowledge that, for example, in the United States alone over 200,000 LGBTQIA+ people, including over 14,000 transgender individuals, living with ACHD.

LGBTQIA+ individuals are also more likely to experience other challenges to their well-being including an increased risk of mental health conditions, an increased risk of suicide, higher rates of tobacco and substance abuse, higher rates of poverty, and higher risks of sexual violence.^62^ Many individuals are reluctant to share their sexual orientation or gender identity with HCPs out of fear of misunderstanding, maltreatment, or simply out of wanting to avoid making the provider feel uncomfortable. Many LGBTQIA+ individuals are very adept at code switching to blend in with cis-gender heterosexual people. ACHD providers have a special opportunity, given the regularity of clinic visits and the longer-term therapeutic relationships, to intervene in specific ways that can help the LGBTQIA+ patient live a healthier life and promote their sexual satisfaction.

There are simple steps HCPs can take to provide a safe environment for LGBTQIA+ patients. Avoiding assumptions when asking about romantic relationships, sexual behavior, and plans for having children and adopting a gender-neutral language can allow individuals to express a preference for their pronouns, discuss their gender identity, or disclose their sexual orientation. For example, asking an individual about a significant relationship removes the gender implications of asking someone if they have a boyfriend or girlfriend. Furthermore, some electronic health record interfaces enable patients to self-report their sexual orientation, gender identity, and preferred pronouns in nonthreatening interfaces.

LGBTQIA+ individuals with ACHD may also have other health conditions that they do not wish to disclose but may affect their cardiac disease. Human immunodeficiency virus infection and its treatment can be associated with cardiac complications. In some countries, the treatment for human immunodeficiency virus and other sexually transmitted diseases are provided outwith the usual health care settings, and therefore the ACHD HCP may be unaware of the potential for drug interactions or other complications. Again, an open and safe environment will increase the opportunities for disclosure.

Nonbinary and transgender individuals encounter many obstacles in obtaining compassionate and competent medical care including legal barriers to gender recognition. Individuals with ACHD have an added layer of complexity when seeking medical or surgical gender-affirming therapies. Transitioning is a very personal and individualized process that usually starts with social transition. Individuals may then seek medical transition, which involves administration of exogenous hormones or hormone-modifying agents. Transgender male patients seeking medical transition are administered testosterone. This does not appear to increase cardiovascular risk though long-term studies are limited. However, testosterone can interacts with several cardiac medications including anticoagulants, SGLT2 inhibitors, beta-blockers, and tolvaptan.^63^

Transgender female patients seeking medical transition are typically given estrogen and antiandrogen therapy. This often includes high-dose spironolactone or GnRH agonists. Estrogen therapies are associated with an increased risk of thrombosis, which may be challenging in those with ACHD. The risk of thrombosis can be reduced by using nonoral preparations. Despite concerns about increased risk of cardiovascular and thrombotic risks, suicide remains the leading cause of death in transgender individuals, which has been shown to decrease with gender-affirming hormone therapy. Therefore, every effort should be made to facilitate gender-affirming care.^64^

Surgical transition may involve chest/breast, genital, or facial reconstruction, as well as electrolysis or laser hair removal. The ACHD health care team can facilitate this in the same way as for other noncardiac surgical procedures. ISACHD will produce a subsequent paper to more fully explore the care of nonbinary and transgender individuals with CHD in conjunction with gender health physicians.

## Cultural considerations

When patients with CHD report a lack of knowledge on sexual intercourse, fertility, and pregnancy, this needs to be done in a culturally competent way. Improving cultural competence among health care workers improves health care quality for culturally and ethnically diverse groups. This results in improved patient satisfaction, treatment adherence, and information seeking.^65^ In many cultures, discussing SA and menstruation are taboo. This can result in complications such as poor reproductive health, poor pregnancy outcomes, and anemia. In communities where sexual relations outside of heterosexual marriage are condemned, this inhibits open discussion and can lead to poor access to contraception and unplanned pregnancies.

One of the major cultural differences that needs to be recognized is the view of personal autonomy. In many cultures, there is a family-centered care model^66^ where the head of the family oversees major health decisions. This lack of personal privacy and bodily autonomy adds complexity to these discussions. One study from Pakistan ^67^ revealed that a majority of parents and teachers thought that age-appropriate sexual education was against religious values, and while education on sexual abuse prevention was largely supported, education on pregnancy prevention was not encouraged. Lack of access to education for girls is also associated with poor reproductive female autonomy ^68^ and lower use of effective contraception. These issues, while affecting the general population may have an incrementally detrimental effect on those with CHD.

Health policymakers and HCPs should find solutions to address cross-cultural differences in sexual health. HCPs should assess the level of autonomy the patient has toward their reproductive health and gauge what concerns them. This level of autonomy will help HCPs take into confidence other people such as partners or even parents. If there is a hesitation by females to express their concerns to a male physician, they should be redirected to a female HCP if possible.

## Individuals with learning difficulties

Although people with CHD attain an average score on intelligence testing, there is growing evidence suggesting specific neurocognitive deficits including gross and fine motor skills, attention, visuospatial ability, speech and language, executive function, social cognition, and impulse control, which may impact sexual health and behavior.

Sexual health is an important determinant of quality of life, including for people with intellectual disabilities. However, these individuals, despite having similar needs for sex and intimacy, have lower levels of knowledge about sexual health.^69^ Disabled persons score lower on sexual knowledge of puberty, reproduction, and sexually transmitted infections. Johoda et al. reported only 1% of patients with intellectual disability in their study had received sexual education.^70^ As a result, these individuals may participate in high-risk behaviors.^71^, have an increased vulnerability to sexual abuse and engagement in inappropriate sexual behavior.^72^ In addition, individuals with intellectual disability may be unable to report a medical condition, which may appear as a problematic sexual behavior to others. Thus, recognition and acknowledgment of the importance of sexual health and education for both the individual and their parents/caregivers are required to empower individuals to develop their sexuality. A crucial issue to address during these conversations is, of course, the individual’s capacity to consent.^73^

Validated tools exist for professionals to assess sexual attitudes, experiences, and needs.^72^ Social and sexual education programs starting before adolescence and extending into young adulthood have proven beneficial in this population.

### How to do things better

#### Normalizing discussions about sexual health

Lack of medical training in sexuality contributes to sexual health being poorly addressed for many people with chronic health problems. In 1 study, individuals with ACHD scored physicians an average of 2/10 on being informed about their experience of sexuality in relation to their ACHD.^21^ A reluctance to broach sexual health may result from attitudinal barriers and assumptions about sex and disability.^74^ The imbalance of power between patient and doctor can also inhibit communication.

**Figure 2:**
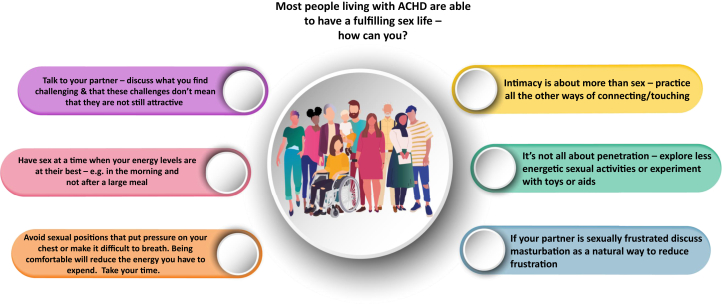
Practical Advice for Those Living With ACHD ACHD = adult congenital heart disease.

The ACHD team has an opportunity to develop a model where psychological safety and trust are communicated through a compassionate approach to care. A named point of contact may improve ease of accessing care (Figure 2).^75^ Normalizing these discussions is essential. Some understanding of trauma informed care will also reduce the risks of difficult consultations or retraumatizing individuals who are vulnerable in this area (Table 3). Again, making assumptions about individuals’ wishes may be detrimental. For many individuals, sexual well-being is not an important part of their lives and medical concerns.

**Table 3:** First Line Assessment for the Cardiac Health Care Team

Exploratory questions
Is your heart condition impacting on any other areas of your life?
How is your relationship doing?
How is your partner dealing with your health issues?
More specific questions
Any sexual or relationship problems?
Has your ability to enjoy sex changed?
Are you scared to have sex?
Baseline assessment
Assessment of physical fitness and symptoms
Plan for optimizing cardiac function
Review medication
Lifestyle issues
Explore psychological well-being
Consider further investigations and/or expert referral

#### Transition

Health care transition is the process of gaining independence with managing one’s own medical care and integrates 2 objectives: the transfer of medical care from pediatric to adult health care professionals and the transfer of medical responsibility from parent to child.^76^

Transition coincides with adolescence already a period marked by psychosocial concerns, sexual development, an increase in risk-taking behaviors, and disengagement with health care services. For these reasons, transition should be seen as an opportunity to engage in preventative health care including discussions centered on sexual and reproductive health. Although not the sole focus of a sexual health history, it is important that adolescents are given the information they need to make healthy choices regarding contraception. A comprehensive transition program empowers and equips adolescents with the tools to discuss scars, body image, and other components important to them in developing a healthy approach to sex and relationships.

#### Educational resources

In some circumstances, patients may feel uncomfortable about accessing information on sexual health directly from their ACHD provider. The use of a curated collection of websites and written material may be less challenging. As with pregnancy and contraception, ACHD services should be producing high-quality, inclusive, and cardiac-specific information. Table 2 lists some of the currently available resources.

#### Expert referral

As previously stated, the ACHD team often serves as the gateway to other specialty services including gynecologist, urologist, endocrine team, and psychology. Many barriers to sexual wellbeing are treatable, and all individuals, including those with ACHD, should not be denied a full assessment and comprehensive care plan. Not all referrals to ‘experts’ relate to health care professionals. The importance of utilizing peer-to-peer support, patient groups, and charities should not be underestimated.

#### Advocacy

There has been a tidal shift in sexual health policy over the last decade. Most important is the linking of sexual and reproductive health rights (SRHR) to human rights.^77^ It is now accepted that sexual health goes beyond pregnancy and contraception, which many ACHD guidelines have focused on, in favor of a more holistic definition as a “state of complete physical, mental, and social well-being in all matters relating to the reproductive system”. SRHR is too often framed as a women’s issue. Modern definitions acknowledge men’s needs as well as the role men can and should play in supporting women’s rights and access to health services. Contemporary definitions also highlight the needs of people of diverse gender identities and sexual orientations with an increasing number seeking care in ACHD centers worldwide.^78^ ACHD programs are well positioned to develop policies and resources that help those living with CHD overcome barriers at the individual, interpersonal, community, and societal levels. Potential barriers include social exclusion, stigma, disability discrimination, and gender-based violence. The World Health Organization,^79^ United Nations Population Fund,^80^ and the Guttmacher-Lancet Commission on SRHR, all provide resources in this area.^78^

#### Improving data collection

There is a need for credible research into the sexual health and well-being needs of adults with CHD. ACHD community should be identifying research priorities that translate into meaningful improvements in the sexual and reproductive health, which is a right frequently under attack.

**Figure 3:**
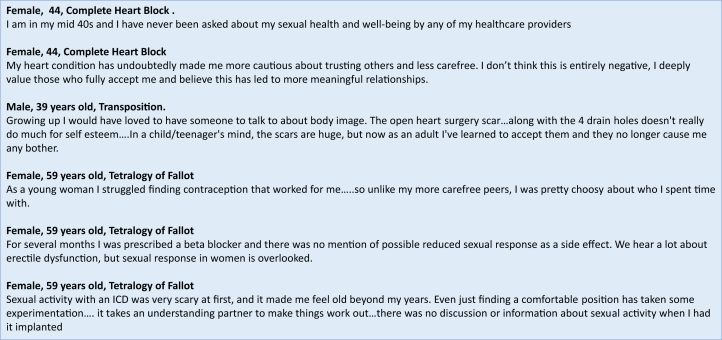
Strategies to Improve Sexual Health and **Well-being**

In conclusion, as the outcome for individuals with ACHD improves, the focus of care needs to move from reducing mortality to facilitating well-being in its fullest sense. Providing the resources for HCPs and those living with CHD to be able to discuss sexual well-being is another step toward a holistic approach to quality of life (Figure 3).

## Funding support and author disclosures

The authors have reported that they have no relationships relevant to the contents of this paper to disclose.
